# Dyke-Davidoff-Masson Syndrome: A Classic Case Report

**DOI:** 10.7759/cureus.34570

**Published:** 2023-02-02

**Authors:** Suruchi Dhawan, Nitin R Rathod, Avinash Dhok, Kajal Mitra, Rushabh Chordiya

**Affiliations:** 1 Radiodiagnosis, NKP Salve Institute of Medical Sciences and Research Centre, Nagpur, IND

**Keywords:** dyke-davidoff-masson syndrome, seizures, magnetic resonance (mr), computed tomography, hemi-cerebral atrophy

## Abstract

Dyke-Davidoff-Masson syndrome (DDMS) is a rare neurological disorder found in children as well as adults. It is characterized by hemi cerebral atrophy. To date, very few cases of this disorder have been reported. Radiological imaging including magnetic resonance imaging (MRI) and computed tomography (CT) are accurate tools for the diagnosis of DDMS. We present a case of a 13-year-old female child who came with complaints of multiple episodes of generalized tonic-clonic seizures. In our case, clinical history and imaging with CT and MRI were accurate enough to diagnose DDMS.

## Introduction

Dyke-Davidoff-Masson syndrome (DDMS) is an uncommon neurological disorder and is characterized by hemicerebral atrophy that typically results from brain injury during fetal or early childhood. It is most often accompanied by contralateral hemiparesis and ipsilateral compensatory osseous hypertrophy [1]. We describe a 13-year-old female child who presented with generalized tonic-clonic seizures and weakness involving the right upper and lower limbs. Neuroimaging results depicted cerebral hemiatrophy, left frontal sinus hyper pneumatization, and asymmetric calvarial thickening.

## Case presentation

A 13-year-old female child presented with multiple episodes of generalized tonic-clonic seizures. The patient was a known case of seizure disorder and was on medication for the same. For the past six months, an increase in the number of seizure events was noted. At the age of nine months, the patient suffered an episode of high-grade fever followed by an episode of seizure and weakness in the right upper and lower limbs. The right lower limb weakness improved over the next six months; however, the right upper limb weakness still persisted. The patient was a full-term normal vaginal delivery, with no prior neonatal intensive care unit (NICU) admissions. All the growth milestones were achieved until nine months of age. On neurological examination, the patient was fully alert and was oriented to time, place, and person. Hypotonia was noted in the right upper limb. The patient had right-sided hemiparesis (power: 2/5 in the right upper limb, 4/5 in the right lower limb). All the deep tendon reflexes were absent. The right plantar reflex was absent and the left plantar reflex was flexor. The rest of the systemic examination of the patient was within normal limits.

**Figure 1:**
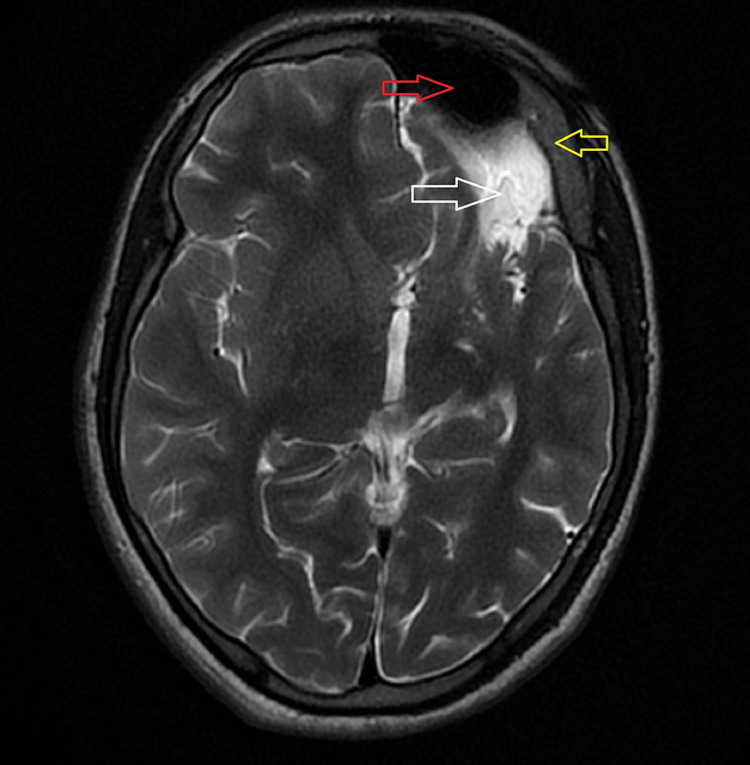
Axial T2 weighted MRI of the brain showing volume loss involving the left cerebral hemisphere (white arrow); Ipsilateral calvarial thickening (yellow arrow); Hyperpneumatization with dilatation of frontal sinus (red arrow). MRI: magnetic resonance imaging

**Figure 2:**
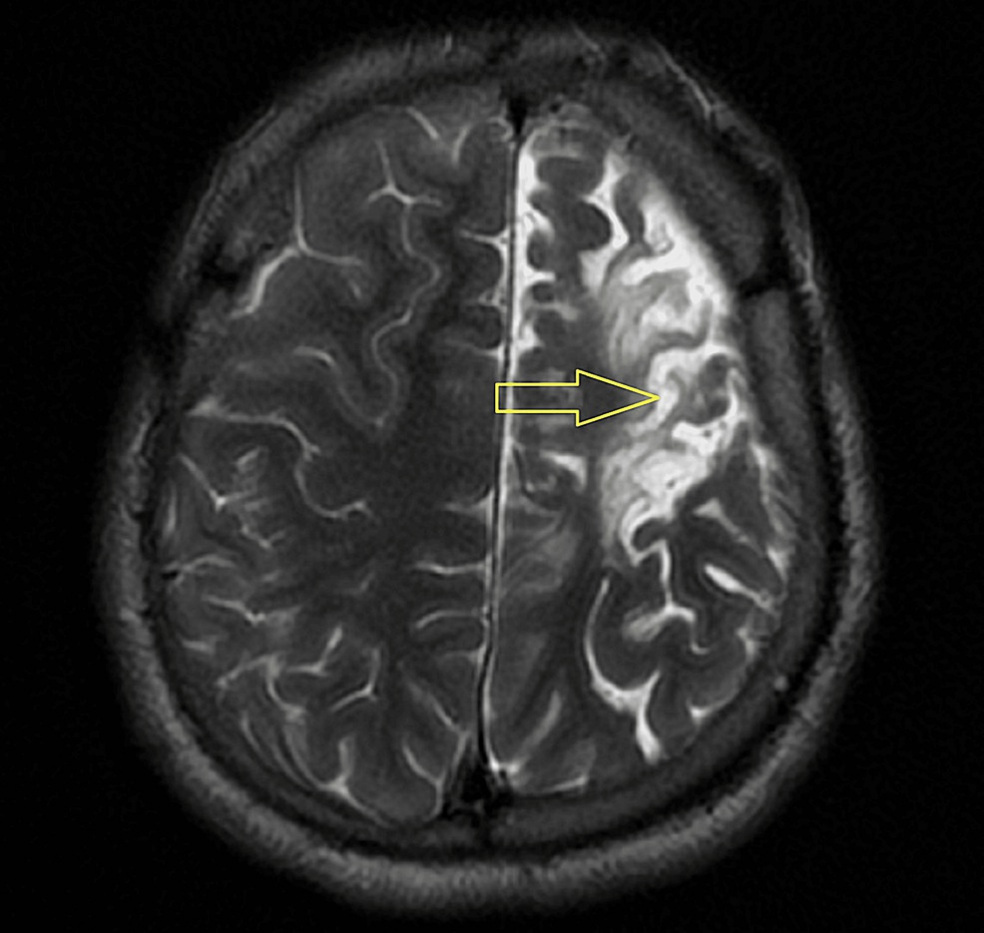
Axial T2 weighted MRI of the brain showing atrophic changes with volume loss involving the left cerebral hemisphere (yellow arrow). MRI: magnetic resonance imaging

The patient has been advised a computed tomography (CT) scan and magnetic resonance imaging (MRI) of the brain. On CT and MRI evaluation, atrophic changes with volume loss and gliosis were noted involving the left frontal and parietal lobes as seen in Figure 1 and Figure 2.

**Figure 3:**
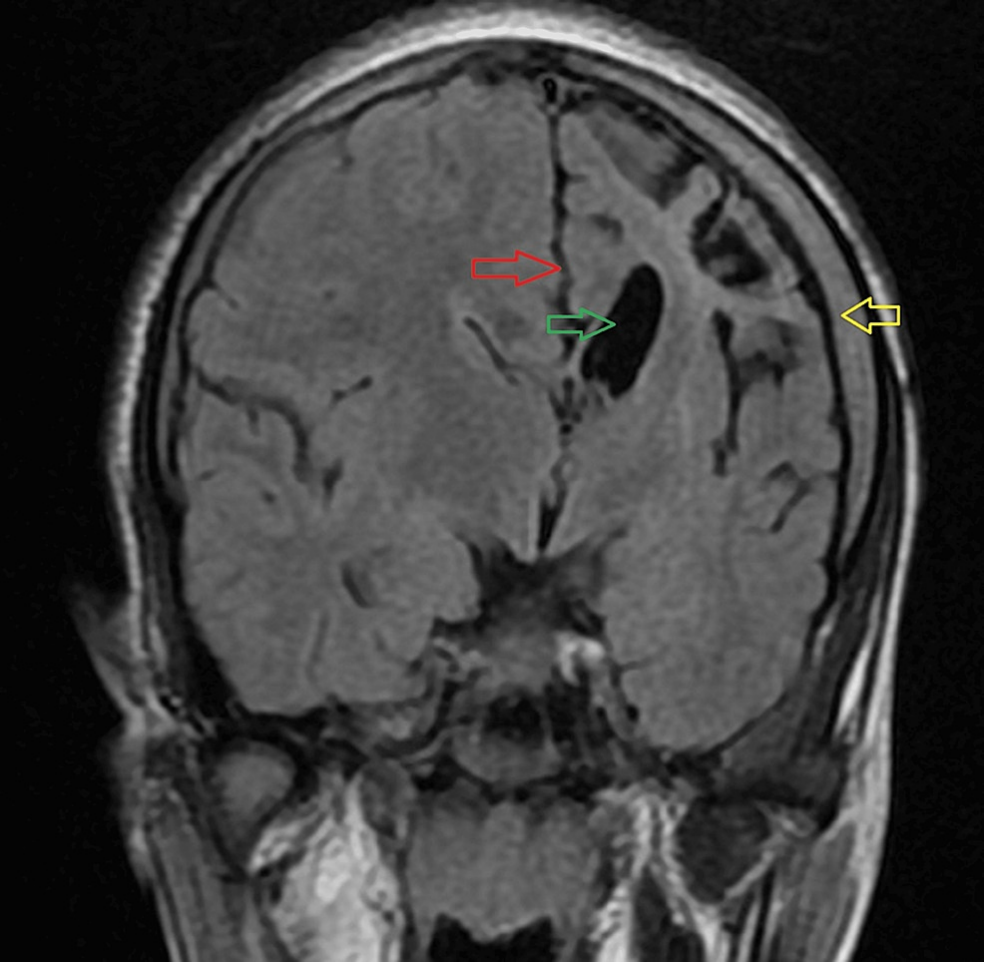
Coronal T2 FLAIR (fluid-attenuated inversion recovery) sequence of MRI of the brain showing calvarial thickening on the left side (yellow arrow) and ex-vacuo dilatation of the ipsilateral lateral ventricle (green arrow). Ipsilateral displacement of the falx is seen (red arrow). MRI: magnetic resonance imaging

**Figure 4:**
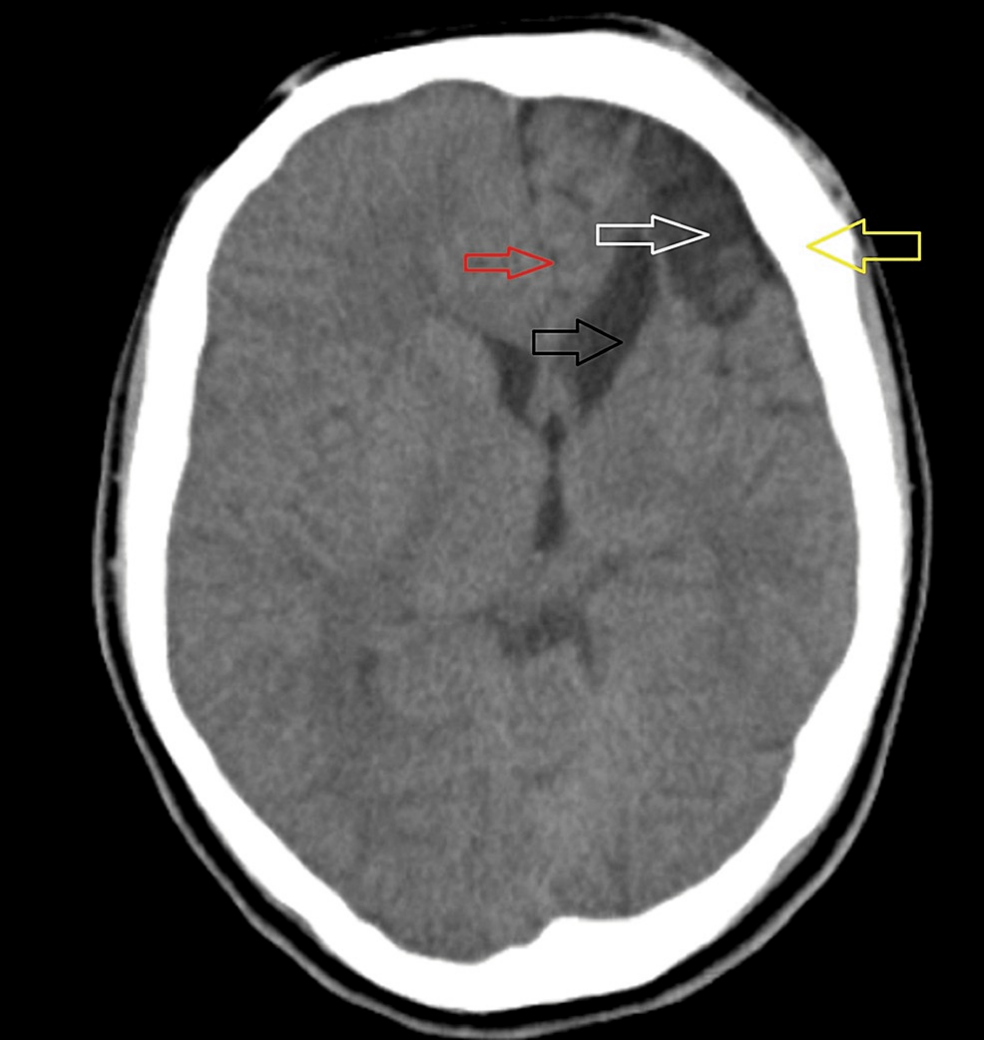
Axial non-contrast CT scan of the brain showing atrophic changes in the left cerebral hemisphere in the frontal region (white arrow), left calvarial thickening (yellow arrow), and ipsilateral lateral ventricle ex-vacuo dilatation (black arrow). Ipsilateral displacement of the falx is also seen (red arrow). CT: computed tomography

This was associated with ex-vacuo dilatation of the body and frontal horn of the left lateral ventricle with the ipsilateral displacement of the falx and other midline structures as seen in Figure 3 and Figure 4.

## Discussion

DDMS was described for the first time in 1933 by Dyke, Davidoff, and Masson on skull radiographs and pneumo-encephalograms [2]. Another commonly used terminology for DDMS is cerebral hemi atrophy. Hypoplasia or atrophy of one cerebral hemisphere in varying degrees accompanied by compensatory changes in the calvarium is known as cerebral hemi atrophy [1]. Half of the normal adult brain size in humans is attained during the first year of life and three fourth of adult brain size is attained within three years [3]. According to Solomon GE et al., brain and outer bony structures of the head develop hand in hand resulting in normal shape and head size, however, if brain development is not appropriate, outer bony size and structure show a compensatory increase in growth causing increased width of diploic spaces, sinus enlargement and raised orbital roof [4]. Sharma S et al. came to the conclusion that improper brain development can be caused by acquired factors such as trauma, infection, vascular abnormalities, and intracranial hemorrhage, which leads to hemi cerebral atrophy and is known as the acquired variant, or by vascular insult or infections during intrauterine life, which results in hypoplasia of a cerebral hemisphere known as the infantile variant [5]. Ono K et al. found that these factors result in several ischemic episodes which further cause a reduction in the formation of brain-derived neurotrophic factors [6].

Clinically, DDMS is described as a syndrome of hemiplegia, seizures, facial asymmetry, and intellectual disability. Seizures can be either focal or generalized [7]. While the symptoms of the infantile variety first appear during pregnancy or infancy, those of the acquired kind first show up throughout puberty or adulthood [8]. In a few cases, there may be ipsilateral Café au lait pigmentations and ocular lipodermoid, contralateral delayed teeth eruption, hypoplasia, and taurodontism [9]. The signs of DDMS may be so minor that conventional radiographs fail to pick them up. CT and MRI are the two gold standard imaging modalities; however, MRI is the diagnostic modality of choice. In 70% of cases, left-sided hemiatrophy is noted. This is likely because the right hemisphere has increased perfusion which is protective in the first three years of life [10].

Imaging findings

On ultrasonography, hemiatrophy and “shifted falx” signs have been reported as early as 29 weeks of gestation. On a plain radiograph, unilateral calvarial thickening with the ipsilateral expansion of paranasal sinuses and mastoid air cells is noted. Most commonly the frontal sinuses are involved since they were pneumatized last and expand up to adolescence. Elevated ipsilateral greater sphenoid wing, petrous ridge, tegmen, fovea ethmoidalis, and planum sphenoidale may also be appreciated on a plain radiograph. On a CT scan of the brain, atrophic changes with volume loss involving the ipsilateral cerebral hemisphere are noted. Enlarged sulci and cerebrospinal fluid (CSF) spaces involving the ipsilateral hemisphere with enlarged lateral ventricle and ipsilateral displacement of the falx are noted. As the patient grows older, these imaging results become more prominent. Compensatory calvarial hypertrophy is often observed in cases of brain injury that occurred during the intrauterine period or prior to the age of three years. In some cases, mild contralateral compensatory hemispheric hypertrophy is seen. On MRI, in addition to the CT findings, the etiology of any atrophy may be more clearly evaluated. Areas of hyperintensity on T2 weighted imaging and fluid-attenuated inversion recovery (FLAIR) along with encephalomalacia and gliosis due to vascular or infectious insult may be seen. Wallerian degeneration of the ipsilateral cerebral peduncle and thalamic atrophy may also be noted [11].

Differential diagnosis

Sturge-Weber Syndrome

Although the skull, sinuses, mastoid, and ipsilateral cerebral atrophy may resemble DDMS, other features such as facial port-wine stain, pial angioma, dystrophic cortical calcification, and ipsilateral choroid plexus enlargement help differentiate it from DDMS [12].

Rasmussen Encephalitis

In this case, hemiatrophy mainly involves the frontotemporal, insular, and parietal regions. There may be crossed cerebellar atrophy or diaschisis. Due to this, it is a close differential diagnosis of Rasmussen encephalitis. However, no calvarial features are seen in Rasmussen encephalitis since it occurs in childhood around six to eight years and 10% of them occur in adolescents or adults [13].

Hemimegaloencephaly

In this case, there is a hamartomatous overgrowth of the cerebral hemisphere with the ipsilateral enlarged lateral ventricle, thereby causing apparent atrophy of the contralateral hemisphere [14].

Parry Romberg Syndrome

In this case, there is typical progressive hemifacial atrophy. Other features include cerebral hemiatrophy, microhemorrhages, calcification, white matter abnormalities, cortical dysgenesis, meningeal thickening, and enhancement [15].

Treatment

Symptomatic treatment is given in the form of antiepileptic drugs for seizures. However, in case of intractable seizures, hemispherectomy is advised. Long-term supportive management in the form of physical, language, and occupational therapy may be required.

## Conclusions

DDMS is a progressive neurological disorder with infantile as well as adult variety. Very few cases of DDMS have been described in the literature till now. A generalized tonic-clonic seizure is the most common presenting symptom of this disorder with other symptoms of brain insult which may progress over time. Therefore, timely diagnosis is essential to initiate the appropriate management. The use of radiological imaging, particularly MRI, enables prompt and accurate diagnosis allowing appropriate management of DDMS.
